# Dislocations of the Shoulder

**Published:** 1893-02

**Authors:** William Meacher

**Affiliations:** Portage, Wis.


### DISLOCATIONS OF THE SHOULDER.

---

BY WILLIAM MEACHER, M. D., PORTAGE, WIS.

---

To the experienced surgeon this may seem to be uncalled for or a repetition of what is to be found in all the text-books on surgery. But I have written this for the ordinary practitioner who has occasionally to deal with a dislocation of the shoulder.

This is the most common of all dislocations and one of the most fruitful sources of malpractice suits, and a large majority of these suits come from the failure of the doctor to discover the lesion. The practitioner who recognizes a dislocation of the shoulder when called to the case, although he fails at reduction, is in little danger of being sued for malpractice. So I feel like attaching rather more importance to the subject of diagnosis, than that of treatment in this paper.

The books on surgery describe a number of varieties of dislocation of the shoulder, namely: downward, forward, backward, and downward and forward—subcoracoid, subclavicular and subspinous—backward on the scapula. But the fact is, as Gross says, that in almost every case the head of the humerus is downward and forward and more or less under the coracoid process. It is rarely in any other position. To have what the books call a subclavicular dislocation there must have been sufficient force to drive the head of the humerus, after leaving the socket, through the flesh or under the pectoral muscle toward the sternum to an unusual extent.

A dislocation directly downward is seldom found, because, should the head of the bone leave the socket in a direction directly downward, the head of the bone would slip forward, more or less into the axillary space, where there would be the least resistance offered to it.

I fear that the student and practitioner is often confused by finding so many varieties of dislocation of the shoulder described; and if each of these varieties could be recognized, the treatment, with very few exceptions, must be the same.

Dislocation backwards, called subspinous, is rare, but is quite as easily recognized as a dislocation downward and forward.

To examine a patient for dislocation of the shoulder, divest him of all clothing down to his waist and do not allow any of the clothing to remain on the sound arm or lie on his lap; take it entirely off him. Seat him on a stool of the same height as a common chair. Do not seat him on a chair if you can provide a stool for him to sit on; the back of the chair will be in your way. Direct the patient to sit upright with his hands resting in his lap close to the abdomen. Now seat yourself on a chair squarely in front of him. If he has a dislocation of the shoulder, you will observe:

1st. That the two elbows do not hang alike; the elbow of the injured arm will be canted out from the body about as much as the thickness of the arm more than the sound one.

2d. The natural mobility of the shoulder joint will be lost to a great extent.

3d. The edge of a yardstick or any straight edge held against the outside of the arm will touch or rest on the outer condyle of the humerus and the acromion process. But if the shoulder is not dislocated, the prominence of the deltoid muscle will hold the straight edge out so that it will not touch the acromion process. The prominence of the acromion is generally very observable, and you can feel a marked difference from that of the sound side. If the patient is a fleshy female, the difference may not show much, but can be plainly felt.

4th. Now feel in the axilla and if the head of the humerus is there instead of in the socket, you can feel it hard and round.

5th. Next, pass a tape measure around under the axilla, and up over the top of the shoulder about an inch in from the point of the acromion process, and if a dislocation exists, you will find that the shoulder measured somewhere in the neighborhood of two inches more in circumference than the sound one. This is one of the most important diagnostic means we possess. Bryant says this was pointed out by the late Mr. T. Galloway in his Jacksonian prize essay in 1849.

6th. Lengthening or shortening of the arm may occur in dislocation, or the length may remain unchanged. If the head of the bone is below the socket and not much inward, the arm will be lengthened. But the head of the bone may be so far inward under the coracoid that the distance from the point of the acromion to the outer condyle of the humerus may be the same as that of the sound arm. Or the head of the bone may be far enough inward under the clavicle to shorten the arm.

7th. Ascertain if the patient can place the hand of the injured arm on the opposite shoulder, while the elbow touches the chest. It is said this cannot be done if the shoulder is dislocated; now, if the patient is a fleshy woman with comparatively small bones and unusually lax ligaments, and has a very full bust, I certainly would not place too much dependence on this test. In male patients, however, and women who are not very fleshy, it is pretty reliable. It is in these fleshy patients, especially fleshy women, that a luxation may be very obscure. And I would strongly advise you in all cases, if you cannot become satisfied without, to place the patient fully under chloroform, and then, if after a thorough examination, you are unable to reach a diagnosis, arrange for further examination the next day, and on several succeeding days, if necessary. A dislocation that is ever so difficult to detect at first, is pretty sure to become apparent before the time passes in which it can be reduced.

A dislocation backward—subspinous—requires a somewhat different description. Looking at the front of the shoulder there will be seen the flattening of the deltoid and prominence of the acromion as in the other form of dislocation, but the arm or elbow instead of being canted out from the side will be close to it, and the arm will be held forward more or less. Looking at the back, the head of the bone resting on the scapula is generally plainly to be seen—a round, hard tumor. An excellent cut of this form of dislocation is shown in Bryant's Practice of Surgery, page 792.

Most of the text-books on surgery direct you to administer an anesthetic to reduce a dislocation of the shoulder, but many of them do not speak positively enough on this subject; and it is astonishing how often reduction is attempted without anesthesia, and so often without success. I believe that one of the reasons why practitioners so often try to reduce shoulder dislocations without anesthetizing the patient is because we still are governed to some extent by the teaching of the days before chloroform and ether. Another reason is that some dislike to put a patient under an anesthetic, but there are very few cases in which reduction should be undertaken without having the patient fully anesthetized.

Many practitioners labor under the impression that a dislocation of the shoulder cannot be reduced without a great deal of pulling; so not frequently the combined strength of two or three men is brought into requisition to pull the unfortunate member, and often without any anesthetic. All this expenditure of force resulting only in torturing the patient. To illustrate this point, I will mention that not many months since, a man came to me saying he had his shoulder dislocated two days ago. He said it had been "put back," but continued to pain him as badly as ever. Upon examination, I found a luxation still existed. When informed of this, he became very much alarmed. He said the doctor who claimed to have reduced it got three men to pull the arm for him and they pulled as hard as they could; the arm itself testifying to his statements by the numerous bruises on it and patches denuded of skin. And he was greatly afraid that his arm would be pulled off if another attempt was made to reduce it, because he supposed that in order to succeed in reducing the dislocation it would be necessary to pull the arm harder than it had been pulled before. But upon being assured that if he would take chloroform, very little if any pulling would be required, he readily consented and the luxation was reduced by manipulation without difficulty.

To reduce a dislocation of the shoulder, place the patient on a firm, level bed, near the edge. Put him fully under the influence of an anesthetic. I much prefer chloroform—Squibb's—because you get more complete relaxation from it.

Reduction by manipulation should be tried first.

It appears to be difficult for writers on this subject to make themselves fully understood in describing the movements of the arm and forearm, which they recommend to be made in trying to reduce a dislocation by manipulation.

Prof. Edmund Andrews, in his excellent treatise in the International Encyclopedia of Surgery, on "injury of the joints" says, "By manipulation is meant a combination of the various passive movements, such as flexion and extension, adduction and abduction, circumduction and rotation by which dislocation of the shoulder can be reduced without the employment of great traction."

I will attempt a little further description of reduction by manipulation.

The patient being fully under the influence of chloroform and lying flatly on his back on the bed with the injured shoulder near to the side of the bed, place yourself at the bedside, a little above the patient's head; grasp the forearm at the wrist, flex the elbow until the forearm is at a right angle with arm, holding the forearm perpendicular, grasp the arm at the elbow with your other hand, and while keeping the forearm perpendicular, abduct the arm as much as you can without using much force. Now rotate it by pulling the forearm from the perpendicular over toward you; that is, in the opposite direction from the patient's feet. Do not use much force in this movement either. Now carry the arm inward over the patient's face, and downward over the chest, at the same time turning the forearm downward toward the patient's feet. Straighten the elbow, thus bringing the arm down close to the patient's side; the last part of the maneuver should be made rather quickly. If this does not succeed after being tried two or three times, then have recourse to Cooper's method—the heel in the axilla. This is probably the most efficient operative procedure the practitioner can adopt. If the dislocation is on the patient's right side, remove the shoe from your right foot; if on his left side, from your left foot; seating yourself on the side of the bed, or on a chair close to it, or, if he lies on the floor (which is as good as lying on a bed, if not better, for this operative procedure,) seat yourself on the floor beside him with your face toward his. Nearly all the works on surgery contain good cuts of this position. Grasp the wrist of the dislocated arm with both hands, place the foot, from which the shoe has been removed, in the axilla, and with the arm at an angle of about 45 degrees out from the patient's body, begin pulling steadily and quite strongly, making counter-extension with the foot in the axilla; after pulling steadily for a short time, (if the bone does not come into place, as it often will by the pulling alone) swing yourself together with the arm toward the patient's body until the arm is brought to the patient's side, continuing the steady pull as you move the arm inward. You will, by this inward movement, pry the head of the bone over your foot out to the socket. This maneuver may have to be repeated several times, but you are almost certain to succeed with it.

As Prof. Andrews says, this procedure, although theoretically faulty, has probably been used in more cases than all other methods combined. It seems irrational, he continues, to pull the head of the bone downward when it is already below the socket. But the secret to success is, the prying out force which is exerted on the head of the bone.

A procedure which might be called a modification of Cooper's method, may sometimes be advantageously employed. For example, in cases where the scapula, from injury, or otherwise is preternaturally movable, or where the head of the bone is driven very far inward under the clavicle.

Place the patient on the floor on his back; when he is fully anesthetized take off both your shoes, and seating yourself on the floor at the injured shoulder, so that when your legs are straightened out they will be at right angles to the long axis of the patient's body, place one foot close up in the axilla, and place the inner edge of the ball of the other foot on the acromion process, (you can make very strong counter-extension on the acromion so,) then taking a firm hold of the wrist of the dislocated member with both hands as in the last described procedure, pull the arm steadily and strongly, keeping it at a right angle with the patient's body, rotating the arm more or less while pulling it.

A dislocation backwards is to be reduced by one or the other of the two last described procedures.

While there is very little danger that one man, in operating, if no mechanical appliance is used and the patient is an adult, will pull hard enough to cause a rupture of the axillary artery or vein, still it is proper to exercise more or less caution in certain cases.

In operating, your hands or the patient's arm may become sweaty and slippery so that you are unable to pull as hard as you need to. In that case, a long towel or a strong strip of sheeting four or five inches wide and of sufficient length, put onto the arm just above the elbow by a clove hitch, will give you the required hold.

Old dislocations, ancient dislocations, and chronic and neglected dislocations are terms applied to cases that have remained unreduced for several weeks or months. Generally, the older a dislocation is, the harder it is to reduce, and *vice versa.*

Generally, after five or six weeks, the chances of reducing a dislocation of the shoulder become limited considerably; even after two or three weeks, some dislocations will be reduced with great difficulty, while others may be reduced, with no more trouble, at the end of six or eight weeks. Cases are recorded of the reduction of shoulder dislocations after the lapse of all lengths of time up to a year and even two years; of course those cases were exceptional.

Very seldom can there be any question about attempting the reduction of a dislocation that is not more than two or three weeks old, but when you come to one which is of six or more weeks standing it always demands very careful consideration.

Some cases it will be best to try to reduce after the expiration of eight or nine weeks, while others it will be advisable to make no attempt to reduce.

Continual pain and suffering from pressure by the head of the bone on the axillary nerves and vessels, with very little motion or usefulness of the member would certainly warrant a good trial at reduction in cases that are eight or nine weeks old, more or less.

On the other hand, if the patient suffered no pain and has a fair amount of motion at the shoulder, as they frequently come to have in course of time, except the overhead motion, so that most of the usefulness of the arm is retained, then it is generally advisable to let the case alone because of the danger to be encountered in trying to reduce old dislocations of the shoulder—the danger of injuring the axillary nerves, and above all, of rupturing the axillary artery and vein. These accidents have occurred a good many times, and a number of patients have lost their lives in this way.

Before operating in old cases, be sure to make the patient fully acquainted with the risk to be taken.

In operating in these old luxations, it is best to employ the first two methods described, manipulation and Cooper's method

—the heel in the axilla; the adhesions about the head of the bone are first to be broken up by manipulation, viz: by rotating the arm quite strongly, first one way then the other, then by abduction and adduction, then rotating again.

A sufficient amount of force can be exerted on the arm by the leverage of the forearm to loosen the head of the humerus from its adhesions in most cases. When you think you have sufficiently broken up the adhesions and loosened the head of the bone, then have recourse to Cooper's method, operating exactly as in a recent dislocation, only you may have to use more force in the pulling and prying it out. It may be necessary to repeat the loosening process several times before you are able to dislodge the head of the bone and place it in the socket.

In the hands of experienced surgeons, the more powerful means may be resorted to when necessary, such as Jarvis' adjuster, (I will not mention the compound pulleys, because they ought not to be used by any one) and laying open the joint by incision and freeing the head of the bone with the scalpel.

